# The Modulation of Exogenous Attention on Emotional Audiovisual Integration

**DOI:** 10.1177/20416695211018714

**Published:** 2021-05-27

**Authors:** Yueying Li, Zimo Li, Aihui Deng, Hewu Zheng, Jianxin Chen, Yanna Ren, Weiping Yang

**Affiliations:** Department of Psychology, Faculty of Education, Hubei University, Wuhan, China; Graduate School of Humanities, Kobe University, Japan; Department of Psychology, Faculty of Education, Hubei University, Wuhan, China; Department of Psychology, Medical Humanities College, Guiyang College of Traditional Chinese Medicine, Guiyang, China; Department of Psychology, Faculty of Education, Hubei University, Wuhan, China; Brain and Cognition Research Center (BCRC), Faculty of Education, Hubei University, Wuhan, China

**Keywords:** emotion, exogenous attention, crossmodal integration, audiovisual stimuli

## Abstract

Although emotional audiovisual integration has been investigated previously, whether emotional audiovisual integration is affected by the spatial allocation of visual attention is currently unknown. To examine this question, a variant of the exogenous spatial cueing paradigm was adopted, in which stimuli varying by facial expressions and nonverbal affective prosody were used to express six basic emotions (happiness, anger, disgust, sadness, fear, surprise) via a visual, an auditory, or an audiovisual modality. The emotional stimuli were preceded by an unpredictive cue that was used to attract participants’ visual attention. The results showed significantly higher accuracy and quicker response times in response to bimodal audiovisual stimuli than to unimodal visual or auditory stimuli for emotional perception under both valid and invalid cue conditions. The auditory facilitation effect was stronger than the visual facilitation effect under exogenous attention for the six emotions tested. Larger auditory enhancement was induced when the target was presented at the expected location than at the unexpected location. For emotional perception, happiness shared the biggest auditory enhancement among all six emotions. However, the influence of exogenous cueing effect on emotional perception seemed to be absent.

In real life, with the developments of technology, the question of whether exogenous attention can facilitate the emotional audiovisual integration has become increasingly more appealing. For example, to enhance the sense of *engagement*, many man–machine interfaces have begun to provide users with emotional audiovisual information. Specifically, video games give players not only auditory emotional feedback but also visual emotional feedback, and players combine this information into a coherent percept, thus enabling a deeper immersive experience than visual or auditory emotional feedback presented alone. Under such circumstances, the following question will arise: Can spatially allocated attention have an effect on emotional audiovisual integration? If the answer is yes, then the emotional audiovisual integration might be facilitated by setting an exogenous attentional cue in interface systems, therefore enhancing the emotional experience of users. Attention can be classified as either endogenous or exogenous. Endogenous attention, also called goal-driven or voluntary attention, involves a more purposeful orienting process driven by an individual’s goals and intentions (Doricchi et al., 2010), for example, orienting to a telephone booth in response to information that your friend is there waiting for you. In contrast, exogenous attention, also called stimulus-driven or involuntary attention, can be understood as the reorientation of endogenous attention to biologically salient events that occur outside of the current attentional focus (Collins & Schirillo, 2013); for example, audiences are attracted by the trick of a magician. In the classic exogenous cueing paradigm (Posner et al., 1980), a bright outline or a rapid flash of light across a potential target location presented on either the left or right side of a computer screen can serve as a peripheral cue to catch an individual’s attention. Improved task performance in terms of rapid detection or better accuracy at the correctly cued location than at the wrongly cued location or uncued location was found (Milliken et al., 2003).

As we pay attention to our social surroundings, information immersed continually in a complex stream of stimulations from multiple sensory channels includes not only unimodal (e.g., visual, auditory, tactile) information but also information from the integration of different modalities. By combining information from different sensory channels, multisensory integration can make better use of the available information and reduce interference in the sensory system (Stein & Meredith, 1990). Evidence shows one-way crossmodal dependence in exogenous orienting from audition to vision (Spence & Driver, 1997) as well as two-way crossmodal dependence in exogenous orienting, whereby audition and vision influence tactile sensation and vice versa (Spence et al., 1998). In addition to multimodal information input, emotional perception, which includes facial expression, gestures, and emotional vocalization, also serves as an inevitable part of human interactions. The integration of emotional signals from the human face and voice ensures the efficiency of emotional recognition and can result in the enhancement of perceptual sensitivity and behavioral responses (Brosch et al., 2008; Miller, 1986), in which the perception of an emotional facial expression can be biased toward the valence of simultaneously presented affective prosodic stimuli and vice versa (Föcker et al., 2011; Rigoulot & Pell, 2012). For instance, fearful and neutral faces were rated as being more fearful when accompanied by fearful sounds than by neutral sounds (Müller et al., 2011). Furthermore, there were evidence showing biases in spatial perception for emotional audiovisual integration (Bertelson & Radeau, 1981), in which visual events always modulate the perceived location of sounds, while sounds produce a much weaker influence on the perceived location of visual events (de Gelder et al., 1999).

However, studies on whether exogenous attention can have an impact on emotional audiovisual integration perception are limited. Most of the research has focused on the relationship between emotion and exogenous attention. In terms of the effects of emotions on exogenous attention, a study applied a color-flanker task to investigate the effects of emotion and color congruency on attentional allocation (Li et al., 2013). In the previous study, congruent processing conditions indicated that the flanker words and the target words had the same color, while incongruent processing conditions had different colors. This study confirmed the effects of emotion on attentional allocation, and such effects varied under different congruency conditions. Furthermore, our study manipulated attentional allocation by presenting a spatially congruent or incongruent cue. Thus, we proposed that the effects of emotion on attentional allocation may also vary in different spatial congruency conditions. Reduced attentional allocation was represented by an enhanced P200-related attentional response to negative stimuli compared to positive stimuli (Carretié et al., 2001). With respect to the modulation of exogenous attention to emotional information, previous studies have shown that exogenous attention can intensify the perception of emotional expressions, and elevated motivation leads to improved efficiency in orienting and reorienting exogenous spatial attention (Engelmann & Pessoa, 2014).

As illustrated earlier, previous studies have demonstrated the interplay between exogenous attention and crossmodal integration, and the biases between different modalities as well as in spatial perception for emotional audiovisual integration. In the past, both scientists and layfolk have considered that the integration of facial and vocal emotional expressions is an automatic process that is independent of attention. This is because we often intuitively, effortlessly, and unconsciously integrate emotional audiovisual information, and previous studies seem to espouse this intuition (de Gelder & Vroomen, 2000; Vroomen et al., 2001). Under such circumstances, the early integration framework was proposed, stating that audiovisual integration is independent of attention (Koelewijn et al., 2010). If that is the case, emotional audiovisual processing should not be affected by the spatial allocation of visual attention. In contrast, the late integration framework asserts that attention is required for audiovisual integration to occur. Evidence challenging the early integration framework has emerged in recent studies, which suggests that the integration of emotional audiovisual information is not independent of attention (Chen et al., 2016; Ho et al., 2015). However, in the previous studies, subjects were instructed to attend to a certain modality while ignoring the other modality; that is, the influence of selective attention on emotional audiovisual integration was tested. Thus, these studies did not solve a critical question, which is whether emotional audiovisual processing can be affected by the spatial allocation of attention; that is, the influence of divided attention (subjects were required to attend to both visual and auditory modalities) on emotional audiovisual integration has not been tested. The current study attempted to address this issue by using a variant of exogenous spatial cueing paradigm, with an exogenous cue presented on the left or right side of the screen that is spatially congruent (valid) or incongruent (invalid) with the target’s location. Facial or vocal emotional target stimuli expressing six basic emotions (happiness, anger, disgust, sadness, fear, surprise) in the visual, auditory, or emotionally congruent audiovisual modality were presented randomly after the cue.

## Materials and Methods

### Participants

Fifty-four participants (20 men and 32 women; aged 18–23 years; mean age = 21.4 years), all right-handed and with normal or corrected-to-normal vision and hearing capabilities, participated in this experiment. To calculate the required sample size, a simple power analysis for a repeated-measures *t* test with a medium expected effect size of dz = 0.5 was performed. That analysis yielded a required sample size of *N* = 54. All participants provided written informed consent to participate in this study, which was previously approved by the Ethics Committee of Hubei University. All participants received payment for their time.

### Stimuli

A black box (1.35 cm × 1.35 cm, with a visual signal of 0.86°) was used as an exogenous attention cue presented on the left or right side of the computer screen and was spatially congruent (valid) or incongruent (invalid) with the target location. Visual emotional stimuli were selected from the Chinese Facial Emotional Pictures collection (Gong et al., 2011). All facial pictures were black and white, 260 mm × 300 mm, and 24 bits. We selected pictures portraying six emotions, including happiness, sadness, disgust, surprise, fear, and anger. For each emotion, there were four pictures (two males and two females). The selection of visual stimuli was based on the dimensions of affective identity and affective intensity. The emotional pictures were chosen to have similar affective intensity scores (approximately 5) and a high identity rate (more than 70%). The rationale of choosing high identity rate pictures was to make sure the facial picture expressed the corresponding emotion, and the choice of similar intensity score pictures was to make sure a similar level of arousal was induced.

For auditory stimuli, students (two males and two females) who were performance artists from Sinan Troupe at Hubei University were asked to say six Chinese interjections (a, ai, ha, yo, ya, o) with happy, sad, disgusted, surprised, fearful, and angry facial expressions. While talking, the speakers were required to generate consistent facial expressions and imitate the expressions of emotional pictures to ensure the validity of the audio stimuli. Before the recording session, the speakers practiced until they could convey the specified emotion with simultaneous facial and vocal expressions. The recording session was conducted in a bright and quiet room, with a smart phone used to record the sound. The samples were standardized to 44.1 kHz, 32 bits, and stereo, with a duration of 500 ms using Adobe Audition CC.

For audiovisual stimuli, auditory stimuli were paired with the facial expressions for each emotion with the same valence and presented in the same direction (left or right). To avoid interactions of speaker sex and emotionality in stimulus pairs, only tokens from same-sex speakers were combined.

### Evaluation Test

In the evaluation test, we tested the self-made auditory stimuli and the retested chosen visual stimuli on affective identity to ensure the validity of all the selected stimuli. Twenty-four college students were recruited to rate the affective identity of previous selected stimuli on a scale of 1 to 9. Each participant needed to complete a session for auditory stimuli evaluation as well as a session for visual stimuli evaluation. The sequence of the previous sessions was counterbalanced across participants. In both evaluation sessions, trials began with a fixation cross presented at the center of the screen for 500 ms; an emotional cue word (anger, disgust, fear, happiness, sadness, surprise) was then presented to suggest the type of emotion that the subsequent stimulus expressed. The duration of the emotional cue word was 1,000 ms, and the subsequent visual or auditory stimulus was presented for 500 ms. Participants needed to report the degree of consistency between the category shown on the screen and the type of emotion that visual or auditory stimuli expressed. Results were showed in Table S1 and Table S2. After collecting the consistency score of each stimulus, one-sample *t* tests were conducted to compare the value of the consistency score with 5. The result showed that the consistency score of all the selected stimuli was significantly higher than 5 (all *p*s < .05).

### Procedure

**Figure 1.**
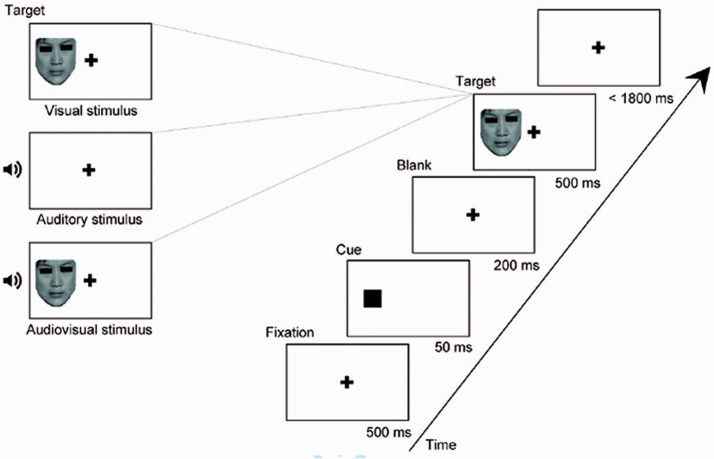
Design of the experimental paradigm.

For the experiment, a 2 cue (valid cue, invalid cue) × 6 emotion (anger, disgust, fear, happiness, sadness, surprise) × 3 modality (auditory, visual, audiovisual) within-group design was employed. The experiment was conducted in a quiet and bright room (Laboratory Room; Hubei University, China). Visual stimuli were generated and displayed with E-prime 2.0 and presented on a monitor (1,680 × 1,050 pixels; 60 Hz). The auditory stimuli were presented by means of two loudspeaker cones located on either side of the computer monitor. Participants sat at a distance of 50 cm from the screen and were asked to complete an emotional discrimination task to identify the emotion portrayed on each trial. At the beginning of each trial, a black fixation point appeared on a white background screen for 500 ms, and then a black box appeared (at a visual angle of 3.4° to the left or right of center) that was spatially congruent (valid) or incongruent (invalid) with the target’s location for 50 ms before target presentation (Figure 1). Then, participants were asked to press corresponding keys to make a judgment on the type of target with the buttons “a,” “s,” “d,” “j,” “k,” and “l,” matching anger, disgust, fear, happiness, sadness, and surprise, respectively. The buttons “a,” “s,” and “d” were pressed by the left hand, and the buttons “j,” “k,” and “l” were pressed by the right hand. A maximum of 1,800 ms was available for responding; after that time, the next trial started. There were 6 blocks, and every block consisted of 144 trials (48 auditory stimuli, 48 visual stimuli, 48 audiovisual stimuli). The proportion of valid and invalid cues was 50% in every modality. It took 40 minutes to perform the task blocks, and the three kinds of stimuli were presented at random to each participant in all blocks. Prior to the formal experiment, a practice experiment was conducted to ensure that the participants were familiar with the key responses.

### Data Analysis

First, to investigate whether the factors (cue validity, emotion, and modality) had a general influence on accuracy (ACC) and response times (RTs), we performed a 2 × 6 × 3 repeated-measures analysis of variance in each presentation condition, with cue validity (valid, invalid); emotion (anger, disgust, fear, happiness, sadness, surprise); and modality (visual, auditory, audiovisual) serving as within-subject factors. Effect sizes are reported as partial eta-squared (${\text{η}}_{p}^{2}$). Analyses of variance were adjusted with the Greenhouse–Geisser nonsphericity correction for effects with more than one degree of freedom. Planned comparisons or post hoc Bonferroni tests were conducted to further explore the interactions between cue validity, channel, and emotion.

Second, to examine the benefit obtained from combining information from the two modalities, we computed both visual enhancement (VE), which reflects the benefit gained from adding an additional visual stimulus to an auditory stimulus, and auditory enhancement (AE), which reflects the benefit gained from adding an additional auditory stimulus to a visual stimulus. The measures of VE and AE have been widely used in investigation of audiovisual performance (Sommers et al., 2005; Sumby & Pollack, 1954; Tye-Murray et al., 2007; Winneke & Phillips, 2011). The formula for response accuracy data was as follows: VE/AE$=\frac{\mathrm{ACC}(\mathrm{visual}/\mathrm{auditory})-\mathrm{ACC}(\mathrm{audiovisual})}{\mathrm{ACC}(\mathrm{audiovisual})}$; and the formula for RT data was as follows: VE/AE$=\frac{\mathrm{RT}(\mathrm{visual}/\mathrm{auditory})-\mathrm{RT}(\mathrm{audiovisual})}{\mathrm{RT}(\mathrm{audiovisual})}$. By conducting the analysis, we could compare the visual contribution to emotional audiovisual integration with or without a valid visual cue, thus addressing the question of whether exogenous attention elicited by a visual cue could facilitate emotional audiovisual integration.

Finally, to examine whether the RTs obtained under the crossmodal condition exceeded the statistical facilitation predicted by the race model (Raab, 1962), the amount of race model violation was calculated under exogenously invalid and valid conditions. The race model is said to be violated when the probability (*p*) of a particular RT is higher in the multisensory condition than the joint probability of the unisensory responses, that is, *p*(AV) > *p*(A + V) – *p*(A*V), for that given RTs. Significant violations of the race model (i.e., RT_AV_ < RT _Race model_) indicate multisensory integration.

## Results

### ACC and RTs

**Figure 2.**
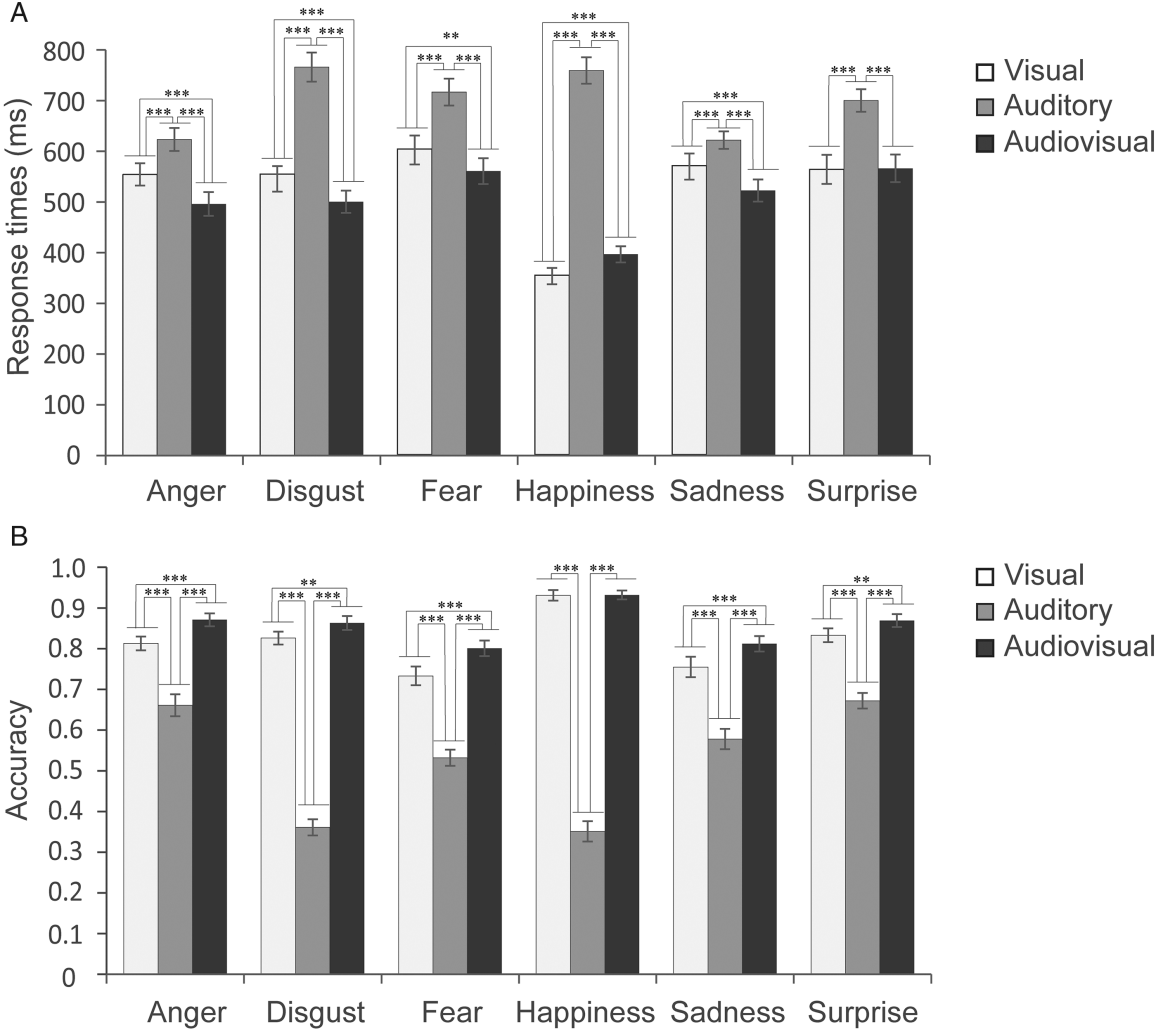
Response times and accuracy for each emotion category with respect to visual, auditory, and audiovisual modality. Error bars represent the standard error (SE). Asterisks indicate significant differences between presentation conditions (**p < .01, ***p < .001).

ACC and RTs for all conditions are presented in Table 1. The analysis revealed a significant main effect of modality on ACC, *F*(2, 106) = 517.889, *p* < .001, ${\text{η}}_{p}^{2}$ = 0.925, and RTs, *F*(2, 106) = 187.661, *p* < .001, ${\text{η}}_{p}^{2}$ = 0.878, indicating that participants responded more effectively under crossmodal condition than under either unimodal condition (all *p* < .001). Importantly, we found significant main effect of cue validity on ACC and RTs. Participants performed less accurately and more slowly under invalid cue than valid cue—72.7 ± 1.4% versus 73.8 ± 1.4%, *F*(1, 53) = 8.908, *p* = .004, ${\text{η}}_{p}^{2}$ = 0.144 for ACC; 578.95 ± 20.27 ms versus 570.11 ± 19.78 ms, *F*(1, 53) = 18.348, *p* < .001, ${\text{η}}_{p}^{2}$ = 0.257 for RTs—which showed the processing advantage of cue validity. There was a significant main effect of emotion on ACC, *F*(5, 265) = 22.265, *p* < .001, ${\text{η}}_{p}^{2}$ = 0.296, and RTs, *F*(5, 265) = 12.308, *p* < .001, ${\text{η}}_{p}^{2}$ = 0.557. Furthermore, main effects were accompanied by two-way interactions between modality and emotion on ACC, *F*(10, 530) = 25.921, *p* < .001, ${\text{η}}_{p}^{2}$ = 0.855, and RTs, *F*(10, 530) = 21.938, *p* < .001, ${\text{η}}_{p}^{2}$ = 0.833. No significant interactions were found between modality and cue validity on ACC, *F*(2, 106) = 0.550, *p* = 0.509, ${\text{η}}_{p}^{2}$ = 0.010, and RTs, *F*(2, 106) = 1.495, *p* = 0.234, ${\text{η}}_{p}^{2}$ = 0.054; between emotion and validity on ACC, *F*(5, 265) = 1.591, *p* = 0.163, ${\text{η}}_{p}^{2}$ = 0.029, and RTs, *F*(5, 265) = 1.368, *p* = 0.253, ${\text{η}}_{p}^{2}$ = 0.122; or between cue validity, modality, and emotion on ACC, *F*(10, 530) = 1.120, *p* = 0.345, ${\text{η}}_{p}^{2}$ = 0.021, and RTs, *F*(10, 530) = 1.125, *p* = 0.341, ${\text{η}}_{p}^{2}$ = 0.021. Post hoc comparisons (see Figure 2) further revealed significantly higher accuracy and faster RTs for bimodal audiovisual stimuli than for unimodal auditory stimuli and for unimodal visual stimuli than for unimodal auditory stimuli in six emotional conditions (all *p*s < .001). The accuracy for audiovisual stimuli was significantly higher than that for visual stimuli in anger (*p* < .001), disgust (*p* = .001), fear (*p* < .001), sadness (*p* < .001), and surprise (*p* = .001) emotional conditions. The RT for audiovisual stimuli was significantly faster than that for visual stimuli in anger (*p* < .001), disgust (*p* < .001), fear (*p* = .003), happiness (*p* < .001), and sadness (*p* < .001) emotional conditions. Happiness induced the highest accuracy and the fastest RTs for visual and audiovisual stimuli among all six emotions.

**Table 1.** Mean Accuracy (%) and Response Times (ms) of Emotion Judgment Based on Visual, Auditory, and Audiovisual Stimulus Modalities Under Valid and Invalid Cue Conditions Across Emotion Categories.

		Mean accuracy (%)					
		Anger	Disgust	Fear	Happiness	Sadness	Surprise
Valid cue	Visual	83.3 (1.7)	83.3 (1.6)	74.2 (2.2)	93.0 (1.1)	76.3 (2.6)	83.0 (1.8)
	Auditory	65.6 (2.8)	37.9 (2.2)	53.7 (2.2)	35.6 (2.6)	58.7 (2.6)	67.3 (2.0)
	Crossmodal	88.2 (1.6)	86.7 (1.8)	80.3 (2.1)	92.6 (1.5)	82.2 (2.0)	86.9 (1.6)
Invalid cue	Visual	79.2 (1.9)	81.8 (1.8)	72.3 (2.5)	93.1 (1.5)	74.7 (2.6)	83.6 (1.7)
	Auditory	66.6 (2.9)	85.9 (2.0)	52.6 (2.1)	34.4 (2.7)	57.0 (2.6)	66.9 (2.0)
	Crossmodal	85.9 (1.7)	85.8 (1.9)	79.9 (1.9)	93.9 (0.9)	80.1 (2.0)	86.9 (1.8)
		Response time (ms)					
		Anger	Disgust	Fear	Happiness	Sadness	Surprise
Valid cue	Visual	541.0 (21.4)	527.3 (24.1)	600.9 (30.9)	345.6 (18.1)	565.3 (26.9)	542.9 (29.2)
	Auditory	633.3 (25.6)	758.8 (33.7)	724.2 (28.5)	733.0 (26.8)	622.4 (19.2)	704.3 (23.9)
	Crossmodal	494.5 (25.2)	486.8 (22.2)	540.4 (27.6)	372.8 (17.4)	515.8 (22.8)	552.3 (27.2)
Invalid cue	Visual	567.8 (24.9)	563.9 (27.7)	604.6 (28.4)	361.9 (15.5)	574.6 (26.8)	585.9 (29.4)
	Auditory	613.3 (26.1)	773.5 (29.9)	709.3 (28.1)	785.7 (31.8)	621.8 (21.5)	696.1 (25.2)
	Crossmodal	497.9 (23.3)	514.1 (24.7)	581.4 (25.6)	420.9 (15.9)	529.6 (23.0)	580.6 (28.4)

*Note*. Standard errors are given in parentheses.

### VE and AE

**Figure 3.**
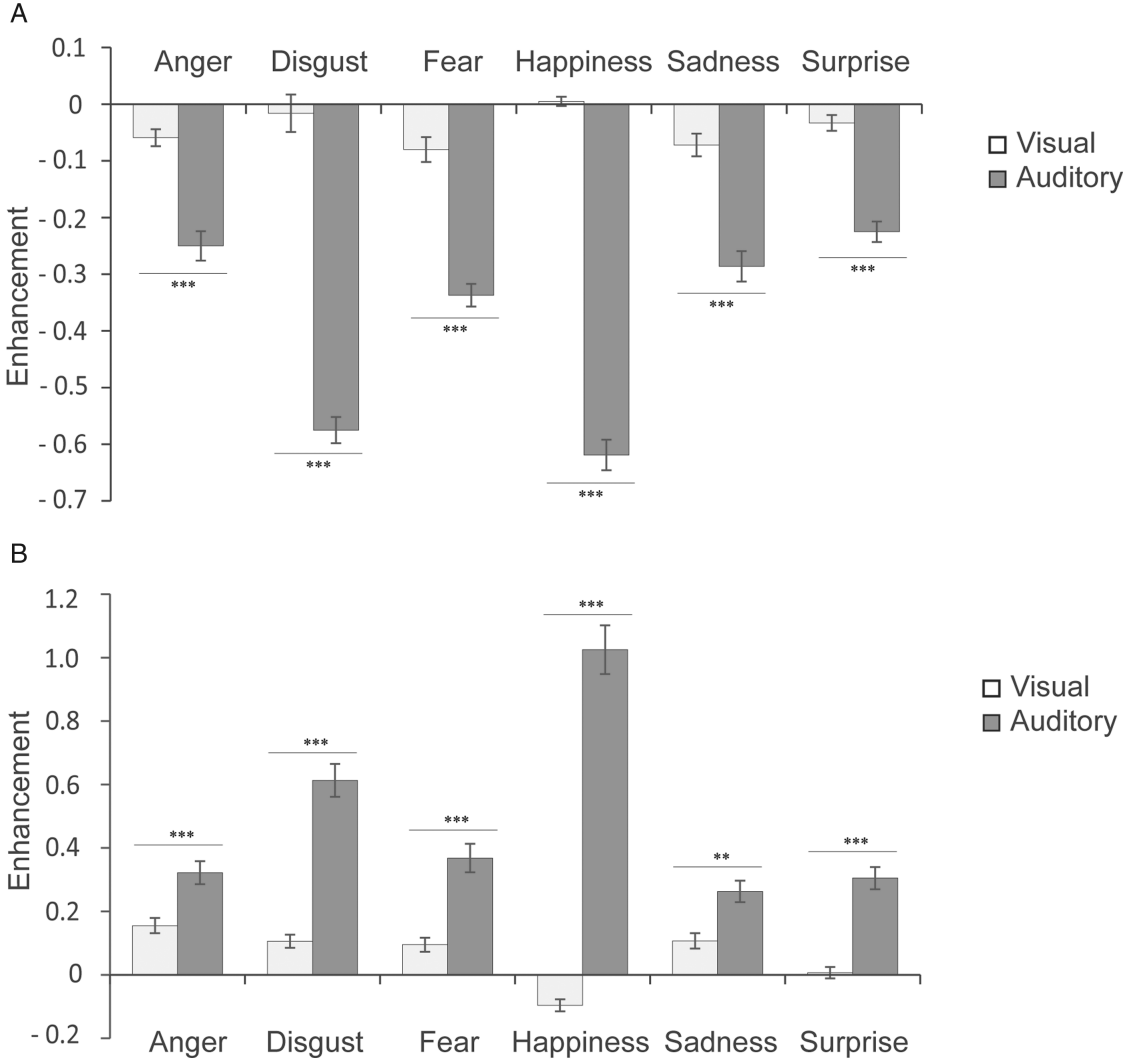
Enhancement scores in accuracy and RTs under each emotion category. Error bars represent the SE. Asterisks indicate significant differences between presentation conditions (**p < .01, ***p < .001).

**Figure 4.**
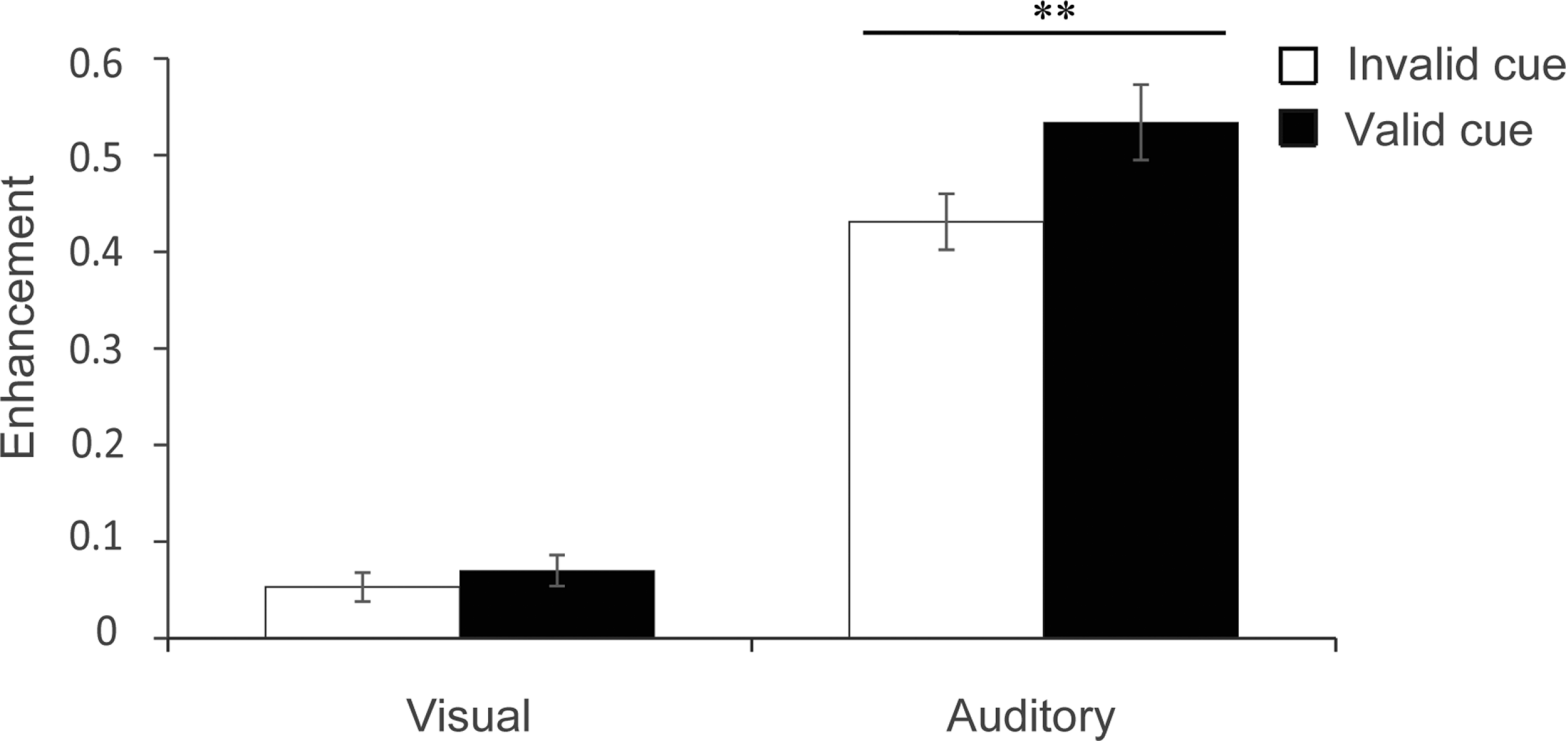
Enhancement scores in RTs with respect to cue validity for all emotion categories. Error bars represent the SE. Asterisks indicate significant differences between presentation conditions (**p < .01).

To demonstrate the level of crossmodal enhancement for accuracy and RTs under exogenous attention conditions, VE and AE were calculated separately. We found significant main effect of modality on ACC, *F*(1, 53) = 405.762, *p* < .001, ${\text{η}}_{p}^{2}$ = 0.884, and RTs, *F*(1, 53) = 188.043, *p* < .001, ${\text{η}}_{p}^{2}$ = 0.78, which indicated larger enhancement for auditory than visual stimuli. Importantly, we found significant main effect of cue validity on RTs, *F*(1, 53) = 8.004, *p* = .007, ${\text{η}}_{p}^{2}$ = 0.131, but not on ACC, *F*(1, 53) = 1.822, *p* = .183, ${\text{η}}_{p}^{2}$ = 0.033. Larger enhancement was induced when the target was presented at the expected location than at the unexpected location. There was a significant main effect of emotion on ACC, *F*(5, 265) = 35.410, *p* < .001, ${\text{η}}_{p}^{2}$ = 0.783, and RTs, *F*(5, 265) = 16.723, *p* < .001, ${\text{η}}_{p}^{2}$ = 0.631. Furthermore, there were significant interactions between modality and emotion on ACC, *F*(5, 265) = 53.691, *p* < .001, ${\text{η}}_{p}^{2}$ = 0.503, and RTs, *F*(5, 265) =33.709, *p* < .001, ${\text{η}}_{p}^{2}$ = 0.775; between modality and cue validity on RTs, *F*(1, 53) = 8.216, *p* = 0.006, ${\text{η}}_{p}^{2}$ = 0.134. No significant interactions were found between modality and cue validity on ACC, *F*(1, 53) = 0.500, *p* = .775, ${\text{η}}_{p}^{2}$ = 0.049, and RTs, *F*(1, 53) = 0.500, *p* = .775, ${\text{η}}_{p}^{2}$ = 0.049; between emotion and validity on ACC, *F*(5, 265) = 1.591, *p* = .163, ${\text{η}}_{p}^{2}$ = 0.029, and RTs, *F*(5, 260) = 1.69, *p* = .137, ${\text{η}}_{p}^{2}$ = 0.031; or between cue validity, modality, and emotion on ACC, *F*(5, 260) = 0.895, *p* = .492, ${\text{η}}_{p}^{2}$ = 0.084, and RTs, *F*(5, 265) = 0.586, *p* = .711, ${\text{η}}_{p}^{2}$ = 0.011. Post hoc comparisons results showed significantly stronger AE than VE for all six emotions for ACC (all *p*s < .05; Figure 3A) and RTs (all *p*s < .05; Figure 3B). Moreover, valid cue can induce stronger AE than invalid cue (0.534 vs. 0.431, *p* = .002; Figure 4). In addition, happiness showed the biggest AE for RT (all *p*s < .001) and ACC (all *p*s < .001).

### Race Model Analysis

**Figure 5.**
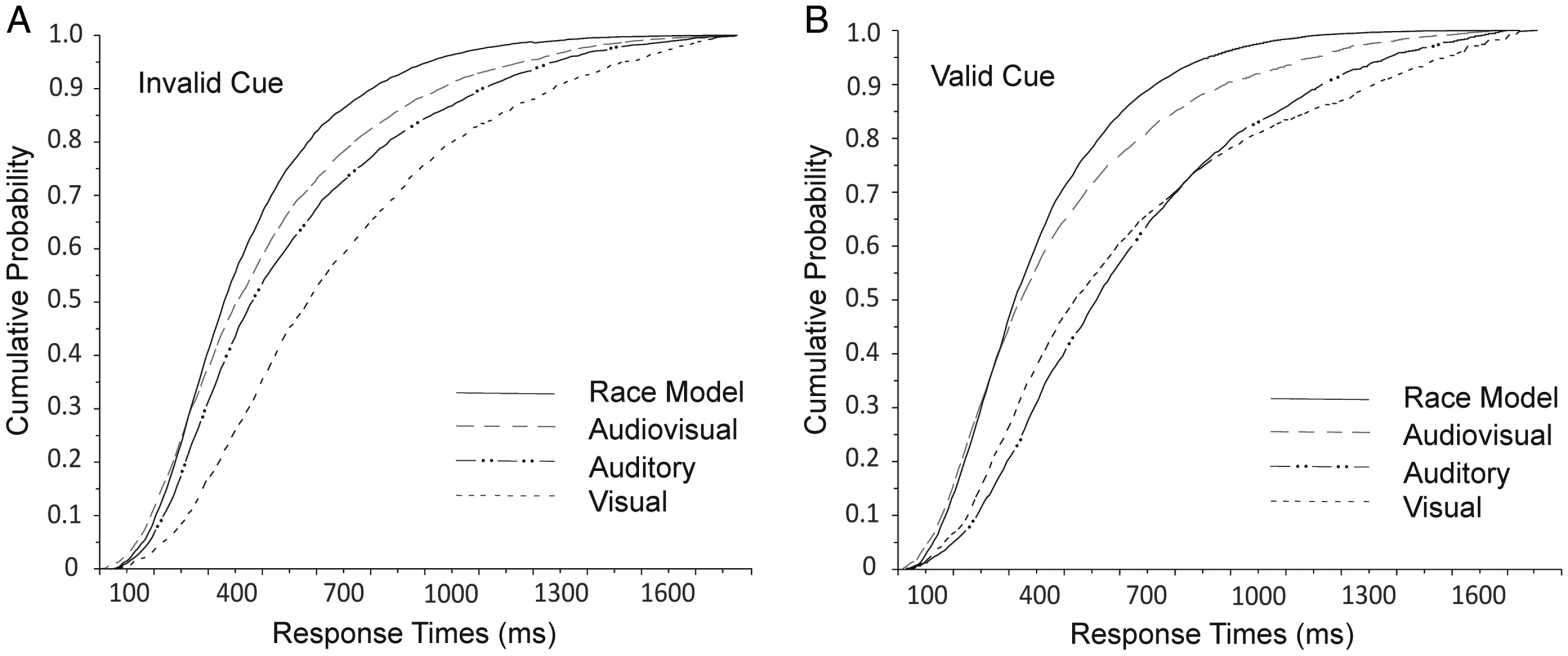
Test for the violation of race model inequality under valid cue conditions (A) and invalid cue conditions (B). The figure illustrates the cumulative probability curves of the RT under the visual, auditory, and audiovisual conditions. The summed probability for the visual and auditory responses is depicted by the race model curve. Note that the crossmodal responses are faster than the race model prediction from 110 ms to 130 ms under invalid conditions and from 100 ms to 120 ms under valid conditions (all *p* < .05).

To test whether race model predictions are met or violated, the RT data were plotted as cumulative distribution functions (CDFs). We divided the RT interval from 90 ms to 1,800 ms into 10 ms bins and calculated the likelihood that a response occurred at a given RT or faster. The CDFs under valid cue conditions and invalid cue conditions are plotted in Figure 5. These data were analyzed by conducting paired *t* tests at each time bin to determine whether the observed audiovisual RT probabilities, *p*(AV), were higher than the joint probability of the unisensory responses, *p*(A + V) – *p*(A * V). The CDF values for RTs to audiovisual conditions were significantly larger (*p* < .05) than the CDF values of the joint probability of the unisensory responses for each time bin from 110 ms to 130 ms under invalid conditions and from 100 ms to 120 ms under validly conditions, which indicated audiovisual integration.

## Discussion

The purpose of the present study was to investigate whether exogenous attention was able to affect emotional audiovisual integration. Visual (facial expressions), auditory (nonverbal affective prosody), and audiovisual (simultaneous, congruent facial, and vocal affective expressions) stimuli expressing six basic emotions (anger, disgust, fear, happiness, sadness, and surprise) were adopted to examine the multisensory nature of emotion processing under exogenous attention. In this study, our results have shown the processing advantage of audiovisual modality over unimodal visual and auditory modalities. We also found higher accuracy and a shorter RT in categorizing emotional stimuli when an onset cue correctly predicts the following target location. Moreover, larger AE was induced when the target was presented at the expected location than at the unexpected location. For emotional perception, happiness indicated the biggest AE among all six emotions. However, exogenous attention and emotional perception seemed to work independently and showed no interactions. One potential limitation of the current study is that the cue we used to attract participants’ attention was a visual cue (a black square); it is possible that the cue worked more effectively for attracting visual attention. Thus, it would be helpful to perform a similar study with an auditory cue in the future. Combination of electroencephalogram or functional magnetic resonance imaging could also be used to further establish the corresponding neural correlates of these effects.

First, we observed higher accuracy and shorter RTs in categorizing audiovisual emotional stimuli than in categorizing unimodal visual or auditory stimuli. Analysis of the race model revealed violations under exogenously invalid conditions (110–130 ms) and valid conditions (100–120 ms), which indicated that the faster responses during audiovisual conditions were likely due to an interaction of the two unisensory information channels and not simply the result of two redundant signals (Gondan & Minakata, 2016; Otto & Mamassian, 2017). The assumptions of independent race model were based on statistical independence between two unisensory signals and context invariance. Compared to unimodal information input, the combination of congruent emotional information from different channels could induce higher response efficiency, which reveals crossmodal integration (Spence & Driver, 1997; Spence et al., 1998). Our results align with previous findings, indicating that emotional judgment tend to improve when more than one source of congruent information about the intended emotion is available (de Gelder & Vroomen, 2000; Massaro & Egan, 1996). The fact that similar results were found by discriminating between fear and disgust emotion expressions displayed auditorily, visually, or audiovisually via short dynamic facial and nonlinguistic vocal clips (Collignon et al., 2008), as well as by presenting stimuli with different combinations of facial, semantic, and prosodic cues conveying five basic emotions (anger, disgust, sad, happy, pleasant surprise; Paulmann & Pell, 2011) provide supportive evidences for the multisensory nature of emotion processing. We then calculate the crossmodal enhancement, which is considered a behavioral marker for crossmodal integration (Calvert et al., 2001), and it features shorter RTs and higher accuracy when participants are confronted with congruent bimodal stimuli than when they are faced with unimodal ones. Our results indicated a stronger AE, as opposed to a VE, under exogenous attention for the six emotions tested, which was in line with previous findings (Maurage et al., 2007), showing that emotion recognition was easier in response to facial stimuli than vocal stimuli (Hunter et al., 2010). As have been illustrated with the visual dominance effect (Colavita, 1974), visual stimuli yielded faster RTs than auditory stimuli. The responses of participants are often driven by the visual stimulus, thus indicating faster RT for visual (e.g., facial expressions, semantic contents conveyed via text) than auditory stimuli (e.g., prosody; Koppen & Spence, 2007; Paulmann & Pell, 2011; Stefanou et al., 2019). Moreover, happiness showed the biggest AE of all six emotions. There is robust evidence that people have attentional bias toward negative emotions (Cisler et al., 2009; Van Bockstaele et al., 2014; Yiend, 2010), which could be regarded as facilitated processing of negative alerting (Asmundson & Stein, 1994; Hope et al., 1990) or difficulty in disengaging attention from negative events (Yiend & Mathews, 2001). In our study, the observed faster RT for happiness under visual and audiovisual stimuli confirms the latter point of view and is in line with previous findings (Laurence et al., 2015) but contrasts with studies (Pell & Kotz, 2011; Rigoulot et al., 2013) using verbal emotional stimuli. It might suggest that nonverbal affective vocalizations are processed at different rates. Researchers have argued that auditory emotion expressions are perceived categorically but in a probabilistic manner over time (Juslin & Laukka, 2003). During emotional communication, auditory and visual events may activate different emotional-related concepts. The physical features which signify emotions in the facial channel can be processed instantaneously and are known to demonstrate strong category boundaries in perception. In contrast, the emotional expressions conveyed through prosody are inherently dynamic and their meanings are unfolded over a protracted time period (Etcoff & Magee, 1992).

Furthermore, we observed faster behavioral responses and higher accuracy for target emotional discrimination in valid than invalid conditions for all manipulations, demonstrating that the allocation of visual attention can be successfully manipulated by an onset visual cue (a black square). Our results indicated the existence of an exogenous cueing effect during the perception of peripheral information and was in accordance with that of Armony and Dolan (2002), who used threaten-related fear stimuli. As suggested by the Posner effect, by shifting exogenous attention to the cue location, the task-irrelevant spatial location of the cue can affect the processing of the subsequent target and modulate the perception of the target (Posner, 1980). If the cue predicts the target validly, there is a benefit in the RT and accuracy. However, if the cue is invalid, a cost occurs in the RT and accuracy (Eimer, 1993). A simple explanation for this effect could be that the valid cue automatically attracts subjects’ visual attention to the location of the target, while the invalid cue attracts their attention to the location of the nontarget areas. Because the ratio of valid cues to invalid cues was 1:1, the effects of expectations were well controlled. In addition, valid cues can augment the significance of spatial orientation to reduce the necessity of spatial orientation to audiovisual targets, thus allowing faster behavioral responses to multisensory stimuli (Brosch et al., 2011). Moreover, the proportion of valid cue conditions can modulate attention in a linear manner; namely, the higher the proportion of valid cue conditions is, the faster the RTs and higher accuracy with valid cue conditions relative to invalid cue conditions (Risko et al., 2008), which is a promising question awaiting future research. In addition to the holistic exogenous cueing effect, we also found stronger AE under valid cues than under invalid cues. Posner (1987) suggested that the presentation of a cue increases alertness and directs attention to that spatial location and enhances the processing of targets in this location. Visual cues can enhance visual target performance, thus making the reaction to visual targets faster than that to auditory targets and inducing stronger AE in audiovisual perception (Ngo & Spence, 2010). Visual exogenous cues that are presented for a short duration give rise to stronger facilitating responding to target stimuli on valid trials than invalid trials and showed the crossmodal facilitation of visual cues to AE. However, the impact of exogenous cueing effect seems to be absent for emotional recognition. Exogenous attention, a bottom-up sensory-driven mechanism that biases selection of stimuli, is thought to operate by involuntarily or exogenously shifting attention to salient stimuli (Collins & Schirillo, 2013). Emotional stimuli are another class of stimuli believed to have the ability to capture attention involuntarily (Ohman & Mineka, 2001). Previous studies indicated that emotional attention and exogenous attention reflect different sources of modulations on sensory processing that operate independently of one another (Brosch et al., 2011). The involuntary capture of attention by emotion-related information involves amygdala (Vuilleumier, 2005), and thus might be at least partly separable from exogenous-attention-related frontoparietal modulation of visual processing (Vuilleumier & Driver, 2007). Although both emotional stimuli and bottom-up exogenously shifting can capture attention involuntarily (Ohman & Mineka, 2001), they may work independently in different brain regions and show no interaction. In addition, by following an involuntary manner, emotion recognition processes may incorporate all available emotion cues, possibly leading to systematically higher accuracy rates as observed here.

## Supplemental Material

sj-pdf-1-ipe-10.1177_20416695211018714 - Supplemental material for The Modulation of Exogenous Attention on Emotional Audiovisual Integration Click here for additional data file. Supplemental material, sj-pdf-1-ipe-10.1177_20416695211018714 for The Modulation of Exogenous Attention on Emotional Audiovisual Integration by Yueying Li, Zimo Li, Aihui Deng, Hewu Zheng, Jianxin Chen, Yanna Ren and Weiping Yang in i-Perception
