# Supplementary material for: The Modulation of Exogenous Attention on Emotional Audiovisual Integration
Source: Iperception. 2021 May 27;12(3):20416695211018714. doi: 10.1177/20416695211018714 (PMC8167015; doi:10.1177/20416695211018714)
Supplement: sj-pdf-1-ipe-10.1177_20416695211018714 - Supplemental material for The Modulation of Exogenous Attention on Emotional Audiovisual Integration [file sj-pdf-1-ipe-10.1177_20416695211018714.pdf]

Table S1 Consistency score of facial pictures（M±SD）

	anger（M±SD）	disgust（M±SD）	Fear（M±SD）	happiness（M±SD）	sadness（M±SD）	surprise（M±SD）
Male face	7.01±0.63	6.05±0.72	6.86±0.92	5.34±0.27	6.97±0.26	6.93±0.80
Female face	7.29±0.48	6.30±0.39	7.49±0.58	5.88±0.32	6.24±0.60	6.97±0.41

Table S2 Consistency score of voice（M±SD）

	anger（M±SD）	disgust（M±SD）	Fear（M±SD）	happiness（M±SD）	sadness（M±SD）	surprise（M±SD）
Male voice	6.73±1.79	7.79±1.19	7.75±1.51	6.33±1.78	6.65±1.71	6.35±1.89
Female voice	6.77±1.90	7.42±1.63	7.52±1.43	7.04±1.49	6.50±2.04	5.88±1.90
